# Notching up a new therapeutic strategy for Non-Small Cell Lung Carcinoma (NSCLC)

**DOI:** 10.18632/oncotarget.671

**Published:** 2012-09-20

**Authors:** Antonio Maraver, Manuel Serrano

**Affiliations:** Spanish National Cancer Research Center (CNIO), Madrid, Spain; Spanish National Cancer Research Center (CNIO), Madrid, Spain

Lung cancer is the leading cause of cancer related death in the world. Current therapies are only partially successful and relapse is frequent in a large number of patients. Hence, new therapeutic strategies are needed to improve the quality of life and survival of NSCLC patients.

The Notch pathway is a key pathway involved in cell fate determination during development in many tissues and also in tissue homeostasis during adulthood [1]. Deregulation of the Notch pathway has been implicated in many human diseases including cancer. Interestingly, the Notch pathway can be oncogenic or tumor suppressive depending on the cellular type. For example, gain-of-function mutations that hyperactivate the pathway are a common cause of acute T-cell lymphoblastic leukemia (T-ALL), while head and neck squamous cancers present loss-of-function mutations of the Notch pathway [1]. In the case of NSCLC, gain-of-function mutations have been found in a small percentage of patients [2, 3] and high Notch pathway activity correlates with poor prognosis [3, 4].

In our recent work [4], we have used a mouse model with a latent genetically-engineered oncogenic *Kras* that can be experimentally induced on adult mice resulting in NSCLC [5]. Importantly, loss-of-function of the Notch pathway completely prevented the generation of NSCLCs [4]. This indicates that oncogenic *Kras*-driven lung carcinogenesis is strictly dependent on the presence of a functional Notch pathway.

**Figure 1:**
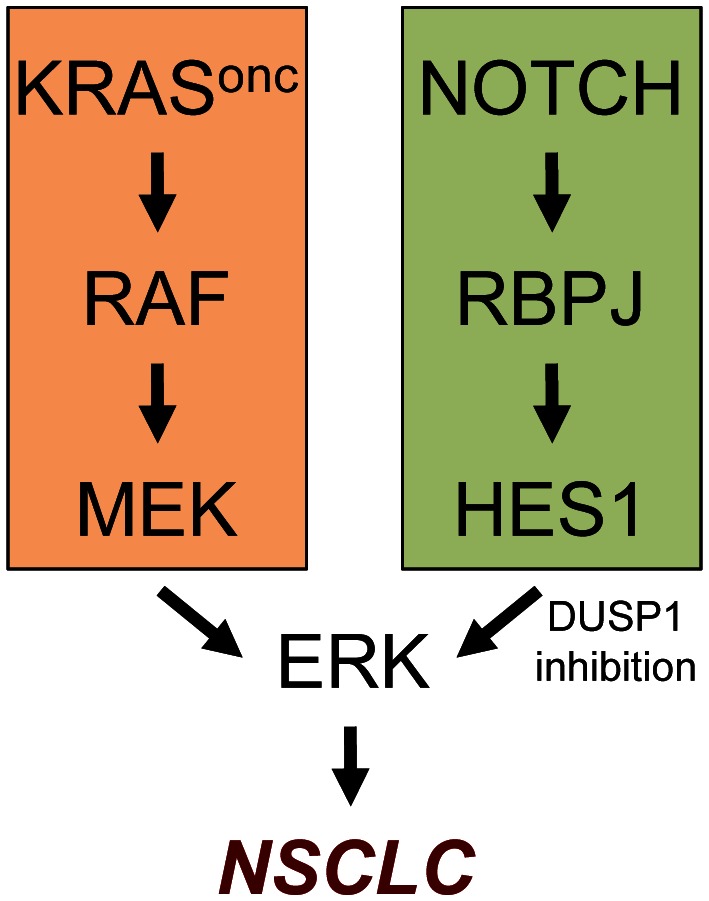
The Notch pathway cooperates with the KRAS pathway in the hyperactivation of ERK in NSCLC

Previous studies have demonstrated that phosphorylated ERK is a critical mediator of oncogenic *Kras*-driven NSCLC [6-8]. Interestingly, our mechanistic studies in murine and human cells revealed that an important, unsuspected, function of the Notch pathway is to contribute to the hyperactivation of ERK [4]. This is accomplished, at least in part, through the transcriptional repression of DUSP1, an inhibitory phosphatase of ERK (Figure 1). Moreover, in primary human NSCLC there is an inverse correlation between Notch pathway activity and DUSP1 levels [4].

Gamma-secretase inhibitors (GSIs) have been used during the last years to inhibit the Notch pathway [9]. Importantly, we found that authochtonous murine *Kras*-driven NSCLC, detected by positron emission tomography (PET), arrested their growth after 15 days of GSI treatment (with Eli Lilly´s GSI LSN-411575). This therapeutic response was associated to biomarkers of Notch pathway inhibition and ERK dephosphorylation [4].

Our results open a new therapeutic opportunity to treat NSCLC using GSIs. Interestingly, GSIs have been used in long-term treatments in Alzheimer´s patients without major side effects (although without improving the course of the disease). The accumulated knowledge on the pharmacology of GSIs should pave the way to test these compounds in NSCLC patients. MEK inhibitors (MEKi) have shown some therapeutic activity in murine *Kras*-driven NSCLC [10]. In this context, combined MEKi and GSIs constitute a very attractive dual agent therapy for NSCLC.
